# Microsatellite instability in Marek’s Disease Virus infected primary chicken embryo fibroblasts

**DOI:** 10.1186/1743-422X-9-193

**Published:** 2012-09-11

**Authors:** Zhenlei Zhou, Dawei Yao, Yan Qiu, Deji Yang

**Affiliations:** 1 College of Veterinary Medicine, Nanjing Agricultural University, Nanjing, Jiangsu 210095, P.R. China

**Keywords:** Microsatellite instability, Marek’s disease virus, Chicken embryo fibroblasts

## Abstract

**Background:**

Marek’s disease virus (MDV), an oncogenic α-herpes virus, causes a devastating disease in chickens characterized by development of lymphoblastoid tumors in multiple organs. Microsatellite instability (MSI), a symptom of defect in DNA mismatch repair function, is a form of genomic instability frequently detected in many types of tumors. However, the involvement of MSI in MDV-infected cells has not been investigated. In this study, we determined the presence and frequency of MSI in primary chicken embryo fibroblasts infected with or without MDV strain *in vitro*.

**Results:**

118 distinct microsatellite markers were analyzed by polymerase chain reaction (PCR) in 21 samples. MSI was found in 91 of 118 markers, and 12 out of 118 demonstrated frequency of MSI at ≥ 40%. 27 of 118 microsatellite loci did not show microsatellite instability.

**Conclusions:**

These findings showed that MSI was a real event occurring in primary chicken embryo fibroblasts infected with MDV *in vitro* as evidenced by the high frequency of MSI, and may be specifically associated with genome alteration of host cells during MDV infected.

## Background

Marek’s disease (MD), caused by the highly oncogenic lymphotropic Marek’s disease virus (MDV), is a highly contagious neoplastic disease of poultry. MD is characterized by lymphomas and lymphoproliferative infiltration of their visceral organs, peripheral nerves, muscles and skin [1]. Although many different kinds of control measures have been implemented against the disease since 1907, MD remains a severe threat to poultry industry in many countries because of its high occurrence. Economically losses from MD is estimated at least US $1 billion annually [2].

The pathogenesis of MD is complex and is not fully understood. Susceptible genotypes of birds are infected by the infectious virus which replicates in the host lymphoid organs. B-cells and macrophages undergo a lytic infection, resulting in the activation of T-cells. The latent T-cells become targets for neoplastic transformation leading to the development of lymphomatous tumors in various visceral organs [3,4]. Some of the factors that lead to MD lymphomas have been investigated. A study demonstrated that MDV might induce host cell DNA strand breaks. Accumulation of genomic alteration over time can lead to gene modifications in cells that might turn to be mutagenic or carcinogenic [5].

Genetic mutations may occur throughout the genome, involving gross chromosomal aberrations or only variations in the length of genomic DNA fragments carrying microsatellite sequences. Microsatellites are repetitive DNA sequences comprised of short reiterative motifs, which locates within the heterochromatin near chromosomal centromeres and telomeres [6]. Microsatellite sequences are stably inherited with high accuracy making them excellent genetic markers. The length of microsatellite sequences is unique to each individual and distinct among individuals. In addition, individual has identical microsatellite in different kinds of cells. Microsatellite mutations or microsatellite instability (MSI) leads to DNA replication error (RER) phenotype. If uncorrected, these errors are fixed after a next round of replication as addition or deletion of one or more microsatellite sequences. This mutated phenotype manifests as MSI, and appears to play an important role in tumorigenesis and/or tumor progression [7-9].

Marek’s disease provides excellent model for the study of herpes virus-induced tumors both in experimental and natural conditions. Therefore the main objective of the present study was to determine the presence and frequency of MSI in chicken fibroblasts infected with the Marek’s disease virus, and to determine microsatellites markers with high frequency of MSI for prediction of host cells gene alteration by virus *in vitro*.

## Results

**Figure 1:**
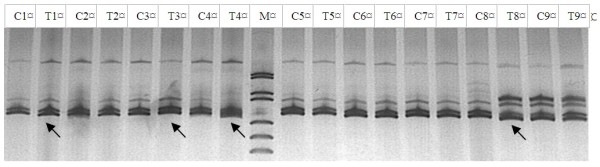
**MSI of MCW0330 in CEFs samples infected by MDV.** Arrows indicated MSI in sample. M: DNA Marker (pUC18 DNA/MspI); C = Control; T = CEFs Sample by virus.

A total of 21 samples were determined for the presence of MIS. All samples were tested at 118 microsatellite loci. Alternations in the size of microsatellites in the MDV-infected samples were revealed as differences in the electrophoretic migration of MDV-infected DNA as compared with control DNA. The MSI frequencies of MDV-infected chicken embryo fibroblasts are summarized in Table 1 and representative samples are illustrated in Figure 1. Twelve markers showed high frequency of MSI (>40%). These markers were ABR0052, ABR0392, LEI0099, ABR0123, ADL0199, ABR0007, ABR204, ABR0086, ABR0059, ABR0634, ABR0133 and ABR0026. 27 microsatellite markers showed no change in length of fragments and 79 microsatellite markers had the lower occurrence of MSI (<40%). Over all, the incidence of MSI in CEF samples of 21 chicken embryos induced by the virus was 100%, at least one microsatellite marker was demonstrated MSI (data not showed).

**Table 1:** MSI frequencies of samples from chicken embryo fibroblasts infected by Marek’s disease virus

**Markers**	**NO. of samples**	**Frequency**	**Markers**	**NO. of samples**	**Frequency**
MCW0248	2,6,11,13,15,17,18,21	8/21(38.01%)	MCW0134	3,4,6,7,9,10,20,21	8/21(38.10%)
ABR0352	1,3,6,8,12,19,20	7/21(33.33%)	ABR0325	4,10,11,14,17,19,20	7/21(33.33%)
ABR0329	5,10,11,12,14,17,18	7/21(33.33%)	MCW0067	2,3,4,8,10,12,14,15	8/21(38.10%)
LEI0209	None	0	ABR0495	3,10,15,17,18,19,20	7/21(33.33%)
ABR0528	3,7,14,16,18	5/21(23.81%)	ADL0038	10,12,13,16,18,20	6/21(28.57%)
ABR0139	3,7,17	3/21(14.29%)	ADL0106	2,9,10,16,18,21,	6/21(28.57%)
ABR0007	2,5,6,7,8,9,10,17,18	9/21(42.86%)	ADL0112	None	0
ABR0379	6,9,11,14,15	5/21(23.81%)	ABR0478	None	0
ABR0518	1,2,6	3/21(14.29%)	ADL0123	None	0
LEI0146	5,6,7,11,16,18,20	7/21(33.33%)	ABR0052	2,4,6,7,9,10,11,14,16,17,19,20	12/21(57.14%)
ABR0525	9,11,12	3/21(14.29%)	ABR0389	6,8,11,12,14,17,21	7/21(33.33%)
ABR0594	4,9,15,21	4/21(19.05%)	ADL0308	1,4,7,10,12,13,14	7/21(33.33%)
ABR0280	None	0	ABR0037	6,11,15,18,19	5/21(23.81%)
ABR0542	4,5,6,7,15	4/21(19.05%)	ABR0059	1,4,7,10,11,12,14,16,21	9/21(42.83%)
ABR0117	1,3,4,9,12,21	6/21(28.57%)	ABR0033	2,3,7,9,11,14,16,18	8/21(38.10%)
ADL0150	None	0	ABR0634	1,2,3,6,9,10,11,14,19	9/21(42.83%)
ABR0368	None	0	ABR0086	1,3,4,6,12,13,14,18,21	9/21(42.83%)
ABR0521	4,9,11,14,17,19	6/21(28.57%)	LEI0099	2,3,4,7,8,9,11,13,14,16,18,21	12/21(57.14%)
MCW0058	4,5,6,8,9,10,	6/21(28.57%)	ABR0271	1,2,6,16,20	5/21(23.81%)
ABR0522	1,6,9,13,19,20,21,	7/21(33.33%)	ABR0506	None	0
ABR0373	None	0	ADL0147	1,2,3,6,9,10,14,21,	8/21(38.10%)
ABR0172	None	0	MCW0322	None	0
ABR0504	None	0	ADL0225	6,11,14,15	4/21(19.05%)
ADL0268	8	1/21(4.76%)	ABR0365	1,6,9,11,14,19,20	7/21(33.33%)
MCW0327	None	0	ABR0517	3,4,5,9,10,12,21	7/21(33.33%)
ABR0247	3,4,10,14	4/21(19.05%)	ABR0530	1,2,6,13,18	5/21(23.81%)
ABR0185	1,3,10,	3/21(14.29%)	ABR0257	None	0
ABR0609	None	0	ADL0293	2,7,9,12,19	5/21(23.81%)
MCW0200	None	0	MCW0330	1,3,4,8,14	5/21(23.81%)
ABR0204	2,6,7,10,13,14,15,18,21	9/21(42.86%)	ABR0387	1,6,8,10,15,17,19	7/21(33.33%)
ABR0649	1,3,10,15	4/21(19.05%)	ADL0199	1,2,3,8,9,13,14,17,18,21	10/21(47.62%)
MCW0036	2,6,10,19,	4/21(19.05%)	ABR0374	3,9,11,14	4/21(19.05%)
ABR0169	None	0	MCW0217	7,8,11,15,16,17	6/21(28.57%)
ABR0284	4,8,12,13,17,19,20	7/21(33.33%)	ABR0650	2,6,7,11,14,16,19	7/21(33.33%)
ABR0113	1,2,6,10,12,16	6/21(28.57%)	MCW0094	None	0
MCW0167	None	0	ABR0133	1,3,5,6,8,13,18,19,20	9/21(42.86%)
LEI0106	4,7,12,17,21	5/21(23.81%)	ABR0180	6,7,9,12,13,16,19	7/21(33.33%)
LEI0168	None	0	MCW0304	8,10,15,19	4/21(19.05%)
MCW0145	3,5,8,9,10,13,15,17	8/21(38.10%)	ABR0364	None	0
ABR0549	4,5,7,8,14,16	6/21(28.57%)	ABR0223	3,18,19,20	4/21(19.05%)
ABR0424	2,6,13,18,21	5/21(23.81%)	ABR0001	7,8,10,11,14,21	6/21(28.57%)
LEI0162	16	1/21(4.76%)	ABR0123	2,5,6,7,8,9,10,11,14,18	10/21(47.62%)
ADL0198	None	0	ABR0026	1,5,6,9,13,14,15,18,21	9/21(42.85%)
ABR0287	4,5,7,8,14,18,19	7/21(33.33%)	ABR0405	2,4,5,6,7,19	6/21(28.57%)
ABR0641	None	0	ABR0624	5,7,15,20	4/21(19.05%)
ABR0631	5,6,10,12,18	5/21(23.81%)	LEI0102	9	1/21(4.76%)
MCW0115	4,7,9,10,11,13,14,20	8/21(38.10%)	ADL0262	17	1/21(4.76%)
ABR0609	None	0	MCW0301	6,15,19,21	4/21(19.05%)
ABR0140	7,8,11,18,20	5/21(23.81%)	ROS0302	3,10,13,15,20	5/21(23.81%)
ABR0392	2,3,4,5,6,7,8,10,12,15,16,20	12/21(57.14%)	MCW0262	1,5,6,11,17	5/21(23.81%)
ABR0046	6	1/21(4.76%)	ABR0109	9,11,18	3/21(14.29%)
ABR0391	1,3,9,10,17,21	6/21(28.57%)	ABR0617	1,6,7,8,9,15,16	7/21(33.33%)
ADL0253	8,11,18,	3/21(14.29%)	MCW0069	8,9,20	3/21(14.29%)
ADL0292	10,19,20	3/21(14.29%)	ABR0006	1,2,3,9,10,18	6/21(28.57%)
MCW0214	4,21	2/21(9.52%)	ABR0015	None	0
ABR0299	11,4,5,6,16	5/21(23.81%)	ABR0076	4,9,13,16	4/21(19.05)
LEI0130	None	0	LEI0135	5,19,20	3/21(14.29%)
ABR0526	4,7,8,10,16,18,20	7/21(33.33%)	ADL0254	None	0
ABR0362	1,4,7,8,14,18	6/21(28.57%)	ABR0066	12,20	2/21(9.52%)

## Discussion

Marek’s disease is a very common disease and provides excellent model for the study of herpes virus-induced tumors in both experimental and natural conditions. In this study, chicken embryo fibroblasts were used to evaluate the effects of MDV on host cells genome *in vitro*. It was reported that not only lymphocytes but also primary chicken embryo fibroblasts could be targets for neoplastic transformation by the MDV [10]. Microsatellite instability (MSI), reflecting a cellular deficiency in DNA mismatch repair (MMR), is now regarded as an important biomarker to predict the cellular genome mutation.

Microsatellites are located in both non-coding and coding portions of the genome, and are thought to play a functional role in gene regulation or indirectly as hot spots for recombination. MSI phenotype mainly caused by mutations in the DNA mismatch repair (MMR) genes. This defect may alter gene expression and contribute to cancer development and progression by accelerating the accumulation of mutations in tumor-related genes [11]. MSI in human tumors development and progression has been extensively investigated, which was regarded to have a closed relationship with malignancies, such as endometrial, renal, colorectal, and gastric cancer [12-15]. The present study indicates for the first time an association between MSI and MDV infection. It was showed that the high frequency of MSI in MDV-infected CEF specimens could be regarded as one of the fundamental feature of CEF transformation. Regulation from oncogenic genes derived from MDV could be a major driving force of the transformation [10]. It is envisaged MDV might lead to defect of MMR system during replication in host cells, which may result in dysregulation of cell division, imbalance between cell growth and death, and act as a trigger for the initiation of cell transformation.

## Conclusions

In conclusion, this study demonstrates that MSI is presented and its frequency is high in MDV infection *in vitro*, and MSI may be related to pathogenesis of MDV disease. In order to identify whether the hypothesis is true, we are investigating and comparing MSI frequency of microsatellites markers in chickens infected with pathogenic strain or the non-pathogenic strains (e.g. CVI988). More studies were still needed to further to determine predictive microsatellite markers and evaluate whether MSI is a useful independent prognosticator.

## Methods

### Chicken embryo fibroblasts (CEF) and Marek’s disease virus infection

Twenty-one chicken embryos (9-days old) from SPF eggs for preparation of chicken embryo fibroblast (CEF) were from SPAFAS Co. (Jinan, China; a joint venture with Charles River Laboratory, Wilmington, MA, USA). Each embryos was trypsinized according to conventional procedures, and the cells were suspended in Medium 199 supplemented with 5% calf serum, glutamine (2 mM), sodium pyruvate (1 mM), Penicillin (1000 u/mL) and Streptomycin (1000 μg/ml) in a 75 cm^2^ flask (Corning Glass Works, Coming, NY). The fibroblasts from each chicken embryo were divided into two for *in vitro* culture. One is assigned for control and another for infection with the virulent RB-1B strain of MDV. The RB-1B strain of MDV was provided by Dr. Aijian Qin (College of Veterinary Medicine, Yangzhou University, China).

### DNA extraction

At day 5 post infection of MDV, 95% of CEF showed cytopathic effect (CPE). The control fibroblasts and MDV-infected CEFs were used for DNA exraction. The cells were scraped from the flasks and pelleted by centrifugation. The pellet was washed once with 0.2 mol/L pH 7.2 phosphate buffered saline and pelleted again. The pellet was lysed in digestion buffer (10 mM Tris–Cl pH 8.0, 0.1 M EDTA pH 8.0, 0.5% SDS, 20 μg/mL pancreatic RNase) and treated with proteinase K. After extraction with phenol, DNA was precipitated with ethanol, dissolved in 1X TE (10 mM Tris–Cl pH 7.5, 1 mM EDTA). The concentration of DNA was determined by OD_260_ using the BioPhotometer plus (Eppendorf, Germany). The quality of DNA was checked by agarose gel electrophoresis.

### Primers, PCR and gel electrophoresis

One-hundred-eighteen microsatellite markers in chicken linkage map [16] were chosen to determine MSI in present study (Table 1). Each microsatellite repeat was amplified by polymerase chain reaction (PCR) and subjected to gel electrophoresis aimed at comparing the pattern of MDV-infected DNA and control DNA from the same chicken embryo fibroblasts. All primers were provided by Dr. Takahashi, National Institute of Agro biological Sciences (NIAS), Tsukuba, Japan.

PCR was performed in a 12.5μL reaction mixture containing 0.5 μl template DNA (100 ng), 0.5 μl of Taq DNA polymerase (2.5 U/μl), 1.25 μl of 10 × PCR buffer, 0.5 μl of each primer (5 μmol/L), 1 μl of dNTPs (2.5 mmol/L), 8.25 μl distilled H_2_O. PCR was performed as following steps: a hot start, 94°C for 15 s, then 94°C for 15 s, 55°C for 30 s, and 68°C for 60 s, all repeated for 10 cycles, followed by 10 under the same condition except for the annealing temperature that was changed to 50°C and 45°C, finally an elongation time of 9 min at 68°C. PCR amplification was performed using the PTC-200 (Bio-Rad, USA). PCR products were separated by electrophoresis in 12% polyacrylamide gel containing 6 M urea for 2.5 h at 70 W. Gels were fixed in 10% acetic acid, and stained with AgNO_3_[17].

### Microsatellite analysis

Each gel was compared with matched normal and MDV-infected CEF specimens. To accurately size the PCR products, a DNA ladder was used on both sides of the gel. Comparisons were made between the number of bands present in both MDV-infected and normal CEF samples. Band intensity was noted but was not categorized for differences between the normal and abnormal DNA. To quantify the presence or absence of MSI, each primer was analyzed for differences in the number of alleles present, be it through insertions or deletions (Figure 1). All microsatellite markers were analyzed for each MDV-infected CEF sample. If the number of allelic bands were not equal in number, band intensity had a significant difference, they were graded as MSI.

## Competing interests

The authors declare that they have no competing interests.

## Authors’ contributions

DJY designed research. ZLZ carried out most of the studies, interpreted the data and drafted the manuscript. DWY and QY participated in parts of the studies and writing. All authors read and approved the final manuscript.
